# Effects of *Lactobacillus acidophilus* on production performance and immunity of broiler chickens and their mechanism

**DOI:** 10.3389/fvets.2025.1554502

**Published:** 2025-03-18

**Authors:** Jinmei Liu, Hongxia Gu, Rui Jia, Shuzhen Li, Zhimin Chen, Aijuan Zheng, Wenhuan Chang, Guohua Liu

**Affiliations:** Key Laboratory for Feed Biotechnology of the Ministry of Agriculture and Rural Affairs, Institute of Feed Research, Chinese Academy of Agricultural Sciences, Beijing, China

**Keywords:** *Lactobacillus acidophilus*, broiler chicken, growth performance, carcass characteristics, immunity

## Abstract

**Introduction:**

*Lactobacillus* species have attracted more and more attention as a potential antibiotic substitute for human health and animal production due to their remarkable antibacterial effects. However, the underlying mechanism is unclear. This experiment’s goal was to investigate the impacts of lactic acid bacteria (LAB) on the growth performance, carcass characteristics, immune function of broiler chickens and their mechanism.

**Methods:**

One hundred and eighty 1-day-old AA broilers were used and randomly allocated into 3 treatment groups with 6 replicates of 10 chickens per replicate. The 3 treatment groups were control group (CK), *L. acidophilus* added group (LAB-E, 1.0 × 108 CFU/kg) for the first 7 days; *L. acidophilus* added group (LAB-A, 1.0 × 108 CFU/kg) for the whole experimental period. Broilers had free access to water and feed.

**Results:**

The results showed that addition of L. acidophilus for the whole experimental period significantly decreased ADFI, FCR and the abdominal fat percentage of broilers (*p* < 0.05), tended to increase the levels of IgG in broiler serum (*p* = 0.093). The LAB-A group had higher HDL-C content and IL-2, IL-4 content, and lower level of LPS in broiler serum compared to the controls (*p* < 0.05).

**Discussion:**

In conclusion, *L. acidophilus* improved feed efficiency and immune function of broilers by controlling nutrient metabolism and inflammation responses of broilers. *L. acidophilus* can be used as a potential substitute for antibiotics in broiler production.

## 1 Introduction

The negative effects of feeding antibiotics on animals, humans and the environment have aroused widespread concern, such as problems with drug residue, antibiotic resistance, and an imbalance in the gut microbiota (1, 2). Finding safe, effective, and environmentally friendly feed additives to replace antibiotics in feed has become a key issue in the development of animal husbandry.

The alternatives include among others prebiotics, enzymes and organic acids, plant essential oils etc. Among them, probiotics are beneficial due to their low cost of production and broad range of use in many host animal types (3–5). The probiotics were finally defined as live micro-organisms that, when administered in adequate amounts, confer a health benefit on the host (6, 7) in 2013 by an expert panel convened by the International Scientific Association for Probiotics and Prebiotics.

Numerous studies have demonstrated that probiotics can regulate the function of the intestinal barriers in poultry (8–11), and positively influence diversity and structure of microbiota, but also nutrient digestibility (12), antioxidant capacity (13) and immune function (14). Probiotic bacteria called *L. plantarum*, which have a wide range of activities, are employed extensively in human and veterinary health as well as the food sector (15). Due to its positive effects on immunology, intestinal barrier function, and cell apoptosis inhibition, *L. plantarum* has the potential to be a widely utilized dietary probiotic (16).

Dowarah et al. (17) isolated an effective probiotic LAB named Lacp from piglet feces and found that supplementation of this probiotic improved FCR compared to control (*p* < 0.001). According to several studies, LAB addition in the diet enhanced broiler performance during the starter phase (18, 19). Olnood et al. (20) found four strains of LAB that altered the gut microflora of birds. Kalavathy et al. (21) discovered that broilers fed a mixture of *Lactobacillus* strains from 1 to 42 days of age demonstrated improved body weight growth (BWG) and feed conversion ratio (FCR), as well as a hypolipidemic impact. Additionally, it has been observed that chickens fed a *Lactobacillus* culture constantly show higher BWG (22). According to the data presented in the paper, LP-8 administration considerably enhanced the broiler growth-related matrix and enhanced immune markers in an industrial chicken farming environment (23, 24). Previous studies on the application of *L. acidophilus* in broiler chickens mainly focused on growth performance, with limited research on the comprehensive effects on production performance, immunity and metabolism. Based on the probiotic properties of *L. acidophilus* and its potential interaction with the gut microbiota, we hypothesized that the addition of *L. acidophilus* to broiler chicken diets would enhance growth performance, boost immunity. We anticipate that *L. acidophilus* can be used as a potential substitute for antibiotics in broiler production. Our study comprehensively analyzes these aspects, aiming to look into how LAB affect the growth performance, carcass characteristics and immune function of broiler chickens and the underlying mechanism.

## 2 Materials and methods

All animal management and experimental procedures for this study were approved by the Animal Ethic Committee of the Chinese Academy of Agricultural Sciences and performed according to the guidelines for animal experiments set by the National Institute of Animal Health.

### 2.1 Birds and housing

One hundred and eighty 1-old Arbor Acres broiler chicks, were randomly assigned into three treatment groups with 6 replicates of 10 chickens per replicate. These 3 treatments were control, LAB-E, LAB-A. The feeding trial lasted for 42 days. Diet (Table 1) and water were provided ad libitum. Bird management followed the Arbor Acre broiler management guidelines.

**Table 1:** Ingredients and chemical composition of experimental diets of broilers (%, as-is basis).

Items	Contents	
	Starter(d 1 to 21)	Grower(d 22 to 42)
Ingredients, %		
Corn	52.59	58.19
Soybean meal	36.18	32.41
Soybean oil	3.76	5.01
Corn gluten meal	3.00	0.00
Dicalcium phosphate	1.72	1.85
Limestone	1.17	1.14
Premix^1^	1.00	1.00
Salt	0.35	0.34
L-Lysine	0.15	0.01
DL-Methionine	0.08	0.05
Total	100.00	100.00
Chemical composition		
ME^2^, MJ/kg	12.55	12.97
CP^3^, %	21.68	19.37
Calcium^3^, %	0.93	0.91
Total P^3^, %	0.71	0.63
Lysine^3^, %	1.06	0.98
Methionine^3^, %	0.48	0.40
Threonine^3^, %	0.86	0.73
Tryptophan^3^, %	0.24	0.22

1 Provided the following per kg of diet: vitamin A, 12,000 IU; cholecalciferol, 2,000 IU; vitamin E (DL-*α*-tocopheryl acetate), 20 IU; vitamin K3, 2.15 mg; riboflavin, 8.00 mg; pyridoxine, 4.5 mg; vitamin B12, 0.02 mg; calcium pantothenate, 26 mg; nicotinic acid, 68 mg; folic acid,1 mg; biotin, 0.20 mg; Fe, 110 mg; Cu, 8 mg; Zn, 78 mg; Mn, 105 mg; I, 0.34 mg; Se, 0.15 mg; choline chloride, 1,500 mg.

2 Calculated values.

3 Analyzed values.

### 2.2 Lactobacillus acidophilus

The *L. acidophilus* was screened from the mixture of fermented silage and soil by the Institute of Feed Research, Chinese Academy of Agricultural Sciences. The strain was identified by the Institute of Microbiology, Chinese Academy of Sciences and deposited in the China General Microbiological Culture Collection Center (CGMCC, address: No. 3, Yard 1, Beichen West Road, Chaoyang District, Beijing City), and the preservation registration number is CGMCC NO. 14437.

### 2.3 Ingredients, diets, and chemical analysis

The ingredients and composition of the experimental diets are shown in Table 1.

### 2.4 Sample collection and analyses

#### 2.4.1 Growth performance and carcass measurements

Chicken body weight (BW) and feed intake (FI) were weekly recorded to recorded to calculate weight gain (WG), and feed conversion ratio (FCR). After chickens’ sacrifice, the FI and BW were recorded, and the average WG and FCR calculated.

On day 42 following 8 h of fasting, all chickens were weighed, and feed intake was measured on a per cage basis. Average daily feed intake (ADFI), average daily gain (ADG), and the feed intake/weight gain ratio (F/G ratio) were calculated. 6 birds per treatment were randomly chosen to analyze the carcass yield. The birds were bled, scalded, and defeathered in a rotary picker. After bleeding, the broilers were defeathered, eviscerated, and discarded the head and feet to determine carcass weight. The abdominal adipose tissue from the surrounding proventriculus and the gizzard down to the cloaca, breast muscle, and legs from each bird were collected and weighed to calculate the carcass characteristics including the dressing percentage, semi-eviscerated percentage, eviscerated percentage, breast muscle percentage, thigh muscle percentage, abdominal fat percentage by the method of Chen et al. (25).

#### 2.4.2 Blood sampling

At 42 days of age, blood samples were extracted from the brachial vein of hens. Following centrifugation (3,000 × g, 150 min), serum was kept for subsequent analysis at −20°C.

#### 2.4.3 Immune organ index

At d 42, 1 healthy chicken per replicate was randomly chosen and humanely killed after fasting 8 h. Thymus, bursa, and spleens were gathered and weighed. The ratio of organ weight to body weight was used to determine relative organ weights.

#### 2.4.4 Immune and inflammatory factors in serum

ELISA kits (Shanghai Enzyme-linked Biotechnology Co., Ltd) were used to measure the amounts of immunoglobulin A (IgA), immunoglobulin G (IgG), immunoglobulin M (IgM), Interferon-gamma (IFN-*γ*); tumor necrosis factor-alpha (TNF-*α*); interleukin 2 (IL-2), interleukin 4 (IL-4) and lipopolysaccharide (LPS).

#### 2.4.5 Biochemical indices in serum

The serum biochemical indices (Urea; UA, uric acid; NH_3_; TC, total cholesterol; TG, triglyceride; HDL-C, high-density lipoprotein cholesterol; LDL-C, low-density lipoprotein cholesterol; VLDL-C: Very low-density lipoprotein cholesterol) were determined using the automatic biochemical analyzer (Model 7,600 Series Automatic Analyzer, Hitachi, Japan).

### 2.5 Statistical analysis

The data was analyzed by a one-factor ANOVA procedure of the SPSS 19.0 software package for Windows (SPSS Inc., Chicago, IL, United States). Duncan’s multiple range test was used to distinguish significant differences between treatment means. The mean and standard error of the mean (SEM) are used to show the results. All statements of significance are based on a probability of *p* < 0.05.

## 3 Results

### 3.1 Effects of *L. acidophilus* on growth performance of broilers

As shown in Table 2, the ADG among the 3 treatment groups were similar. LAB-A significantly decreased the ADFI and FCR of broilers during d1-21 compared with other groups (*p* < 0.001). During the whole period, LAB-A significantly decreased ADFI and FCR (*p* < 0.05).

**Table 2:** Effects of *Lactobacillus acidophilus* on performance of broilers.

Parameters	CK	LAB-E	LAB-A	SEM	*p* value
Initial BW, g	42.88	42.87	42.92	0.203	0.995
Final BW, g	2,370	2,310	2,400	0.030	0.532
Day 1–21					
ADG, g	35.47	34.36	33.71	0.367	0.141
ADFI, g	54.17^a^	52.79^a^	45.93^b^	0.978	<0.001
FCR	1.53^a^	1.54^a^	1.36^b^	0.021	<0.001
Day 22–42					
ADG, g	75.54	73.71	76.20	1.161	0.688
ADFI, g	135.63	130.79	128.59	2.900	0.146
FCR	1.80	1.78	1.69	0.038	0.082
Day 1–42					
ADG, g	55.50	54.03	55.96	0.676	0.695
ADFI, g	94.90^a^	91.79^a^	87.26^b^	1.775	0.022
FCR	1.71^a^	1.70^a^	1.53^b^	0.030	0.015

a, b means without common letters differ at *p* < 0.05.

BW, body weight; ADG, average daily gain; ADFI, average daily feed intake; FCR, Feed conversion ratio.

CK, control group; LAB-E, *lactobacillus* added group (1.0 * 108 CFU/kg) for the first 7 days; LAB-A, *lactobacillus* added group (1.0*108 CFU/kg) for the whole experimental period.

### 3.2 Effects of *L. acidophilus* on carcass characteristics of broilers

As shown in Table 3, the LAB addition group had less abdominal fat percentage of broilers (*p* < 0.05). The other carcass traits, such as dressing, semi-eviscerated, eviscerated, breast muscle and thigh muscle percentage, were not affected by *Lactobacillus* addition.

**Table 3:** Effects of *Lactobacillus acidophilus* on carcass characteristics (%).

Parameters	CK	LAB-E	LAB-A	SEM	*p* value
Dressing percentage	92.21	91.82	91.09	0.179	0.128
Semi-eviscerated percentage	85.20	84.99	84.25	0.259	0.681
Eviscerated percentage	73.61	73.25	72.49	0.763	0.644
Breast muscle percentage	29.55	28.98	28.92	0.578	0.707
Thigh muscle percentage	28.84	33.41	32.29	0.935	0.165
Abdominal fat percentage	1.97^a^	1.60^a^	1.54^b^	0.075	0.035

a, b means without common letters differ at *p* < 0.05.

### 3.3 Effects of *L. acidophilus* on immune organ indexes of broilers

As shown in Table 4, LAB addition did not significantly affect immune organ indexes (spleen, thymus, bursa of Fabricius) (*p* > 0.05).

**Table 4:** Effects of *Lactobacillus acidophilus* on immune organ indexes of broiler.

Parameters	CK	LAB-E	LAB-A	SEM	*p* value
Spleen	3.18	3.38	3.80	0.198	0.691
Thymus	6.24	6.35	6.39	0.207	0.806
Bursa of Fabricis	1.14	1.19	1.33	0.116	0.951

### 3.4 Effect of *L. acidophilus* on immunoglobulin levels of broilers

As shown in Table 5, LAB-A tended to increase the levels of IgG in broiler blood (*p* = 0.093), but it was not significant.

**Table 5:** Effects of *Lactobacillus acidophilus* on immunoglobulin levels of broilers.

Parameters	CK	LAB-E	LAB-A	SEM	*p* value
IgA, g/L	1.14	0.99	1.23	0.051	0.154
IgG, g/L	8.20	8.04	10.35	0.475	0.093
IgM, g/L	0.78	0.70	0.76	0.018	0.185

IgA, immunoglobulin A.

IgG, immunoglobulin G.

IgM, immunoglobulin M.

### 3.5 Effect of *L. acidophilus* on nitrogen content in serum of broilers

As shown in Table 6, LAB addition did not significantly affect the content of UREA and UA, but significantly reduced the content of NH_3_ in serum of broilers (*p* < 0.01).

**Table 6:** Effects of *Lactobacillus acidophilus* on nitrogen content in serum of broilers.

Parameters	CK	LAB-E	LAB-A	SEM	*p* value
Urea, mmol/L	0.705	0.632	0.613	0.049	0.749
UA, μmol/L	320.20	225.67	197.33	24.853	0.119
NH3, μmol/L	426.41^a^	268.68^b^	238.24^b^	28.188	0.005

UA, uric acid.

a, b means without common letters differ at *p* < 0.05.

### 3.6 Effect of *L. acidophilus* on lipid content in serum of broliers

As shown in Table 7, LAB-A significantly increased the content of HDL-C in serum of broilers compared with CK or LAB-E group (*p* < 0.05), but other differences were observed across groups (*p* > 0.05).

**Table 7:** Effects of *Lactobacillus acidophilus* on lipid content in serum of broilers.

Parameters	CK	LAB-E	LAB-A	SEM	*p* value
TC, mmol/L	3.36	2.98	3.00	0.073	0.052
TG, mmol/L	0.62	0.55	0.48	0.030	0.152
HDL-C, mmol/L	2.17^b^	2.16^b^	2.41^a^	0.044	0.027
LDL-C, mmol/L	0.80	0.68	0.70	0.030	0.258
VLDL-C, mmol/L	0.15	0.13	0.12	0.017	0.858

TC, Total cholesterol.

TG, Triglycerides.

HDL-C, High-density lipoprotein cholesterol.

LDL-C, Low-density lipoprotein cholesterol.

VLDL-C, Very low-density lipoprotein cholesterol.

a, b means without common letters differ at *p* < 0.05.

### 3.7 Effects of *L. acidophilus* on inflammatory factors in serum of broilers

As shown in Table 8, the LAB addition increased the levels of IL-4 and IL-2, and reduced the level of LPS in broiler serum significantly (*p* < 0.05).

**Table 8:** Effect of *lactobacillus acidophilus* on the content of inflammatory factors in serum of broilers.

Parameters	CK	LAB-E	LAB-A	SEM	*p* value
IFN-γ, pg./mL	100.99	106.49	114.27	1.981	0.271
TNF-α, pg./mL	73.63	76.69	78.21	1.057	0.225
IL-2, pg./mL	39.63^b^	38.39^b^	49.20^a^	1.898	0.022
IL-4, pg./mL	152.60^b^	174.85^a^	187.31^a^	4.492	0.001
LPS, U/L	40.13^a^	39.64^ab^	35.45^b^	0.760	0.016

a, b means without common letters differ at *p* < 0.05.

## 4 Discussion

Numerous studies have shown that LAB, a predominant group in the intestinal tract, are instrumental in maintaining the balance of the gastrointestinal microbiota. They are essential for safeguarding the integrity of the intestinal barrier, enhancing mucosal immunity both *in vivo* and *in vitro*, and preventing pathogenic infections (26–29). Thus, *lactobacillus* is considered to be a type of probiotic alternative to antibiotics to control and prevent animal’s disease (27, 28)and widely used in the field of livestock and poultry production.

### 4.1 Growth performance and carcass characteristics

Growth performance and carcass characteristics are the most direct indicators of broiler production, which are critical to improving economic performance.

*Lactobacillus* is a typical probiotic, which can colonize the digestive tract of animals, release a range of digestive enzymes, and enhance the environment of the digestive tract through metabolic processes. Moreover, it has the ability to enhance intestinal development by augmenting the villi height - to - crypt depth ratio in the ileum, thus accelerating nutrient absorption, improving feed conversion efficiency, and promoting animal growth (26, 29–31).

Numerous studies have selected and applied LAB to enhance the growth performance of animals. Salehizadeh et al. (32) used the selected mixture of *lactobacillus* and commercial probiotics in their experiments, which had a significant effect on the growth performance and carcass characteristics of broilers.

According to Shokryazdan et al. (33), adding an *L. aalivarius* blend to the ration considerably raised the ADG and feed conversion ratio of broilers. Some studies discovered that, final weight, total weight increase, and average daily growth of broilers fed *L. plantarum* were all significantly higher than those of the control group, according to Humam et al. (34). This might be attributable to the fact that *lactobacillus* produce bacteriocins and organic acids, which inhibit the growth and reproduction of pathogenic bacteria in the gut (35). LAB can colonize the digestive tract of animals and produce various digestive enzymes, including amylase and protease, which help promote the absorption of nutrients and improve the feed conversion rate (26, 36). In the present study, we found that LBA-A significantly decreased ADFI, FCR of broilers during the 1–21D and whole period. These results are similar to those obtained in previous studies (37, 38). It may be beneficial for growth performance improvement. Concerning carcass traits, our results are consistent with the result of Junaid et al. (14) that supplementing *Lactobacillus* did not affect carcass traits in broiler chickens. There is little evidence that probiotics significantly affect carcass percentage and related parameters, such as breast, thigh and drumstick, and wings (39).

### 4.2 Immune function

Thymus and bursa are the central immune organs of poultry, which affect the cellular and humoral immune function of the body. The spleen, the body’s biggest lymphoid organ, is home to a vast population of macrophages and lymphocytes (14), which is both the peripheral immune organ and the site of lymphocytes to settle and respond to antigen stimulation. For example, the growing relative weight of immune organs is an indication that both the cellular and humoral immune functions have been strengthened (26). Vineetha et al. (40) found that weight of immune organs was higher in the *L. plantarum* LGFCP4- supplemented group. Awad et al. (22) found that *lactobacilli* can promote the growth and development of immune organs in chickens. LAB dietary supplementation in this study did not significantly affect the weight of broiler immunological organs. The inconsistency may be attributed to the added dosage of lactic acid bacteria or the small statistical unit-size.

The immunoglobulins play a crucial role in the immune system, thus serum immunoglobulin level is the most commonly used index to measure immune function. IgA is involved in the immune process of mucosal infection, IgG is a reactive immune response antibody with antibacterial and antiviral effects, IgM is involved in the initial immune response and has the same anti-infective effect as IgA (41–44). In the study by Riaz Rajoka et al. (45), LAB strengthened the host’s immune system by boosting the production of IgA and the number of immunological and epithelial cells. In our experiment we found that LAB addition tended to increase the levels of IgG in broiler blood, indicating that *lactobacillus* enhance immune function of broilers. IgG is mostly produced and released by plasma cells found in lymph nodes and the spleen. Vineetha et al. (40) proved that the weight of immune organs was higher in *L. plantarum* LGFCP4-supplemented group. In this study, dietary supplementation with LAB for the whole experimental period tend to increase the immunoglobulin levels of broilers, which may show that the dosage of lactic acid bacteria needs to be further adjusted.

### 4.3 Serum parameters

In poultry, amino acid metabolism is primarily regulated by the purine nucleotide cycle, with uric acid being the major end product. External microorganisms can further break down uric acid into urea through the enzymatic activities of uric acid oxidase and allantoinase. Subsequently, microbial urease facilitates the conversion of urea into ammonia (46). The concentration of urea, a metabolite of protein, in the bloodstream serves as an indicator of the bird’s immunological status. In this study, compared with the control group, both experimental groups, LAB - A and LAB - B, showed a decrease in the levels of uric acid and urea nitrogen in broilers. Moreover, there was a significant reduction in NH_3_ levels in broilers compared to the control group, indicating an effective regulation of nitrogen metabolism in chickens.

Our results showed that the addition of LAB could reduce the content of HDL-C and TC in blood of broilers. LAB were able to assimilate cholesterol significantly in-vitro, Cell wall binding and incorporation of cholesterol within their phospholipid layer was reported as a possible mechanism for cholesterol assimilation (47), and bile salt hydrolytic activity (BSH) of probiotics also stand as the most significant mechanisms for cholesterol removal (48). Broiler triglyceride and cholesterol levels were significantly reduced by both native and commercial LAB, according to Salehizadeh et al. (32). Moreover, Shokryazdan et al. (33) found a similar effects of LAB probiotic strains on serum lipids of chickens. Jeon, Lee, and Chang (49) also confirmed that probiotic LAB (*L. plamtarum* E.M) showed a significant hypocholesterolemic effect and metabolism improvement in rats. While the exact mechanisms are unknown, there are a number of researchers that are focused on either increasing the breakdown and excretion of cholesterol or decreasing its synthesis (50). And another mechanisms were also proposed, some lactic acid bacteria are able to assimilate cholesterol into their cells resulting in cholesterol reduction of surrounding environment (51).

Cytokines are crucial for the inflammatory response and immune system (52). Assessing the production of cytokines is essential for assessing the cell-mediated immune response (53). Proinflammatory (IL-1*β*, IL-2, IL-6, IFN-*γ*, and TNF-*α*) and anti-inflammatory (IL-4, IL-10, and TGF-β) cytokines are the two main categories of cytokines. Proinflammatory cytokines are crucial for the innate and adaptive immune responses to form and function. Pathogens are removed from the host by these immune cells, while their overexpression is linked to pathological immune system disorders and may have detrimental impacts on host growth and health (54). Disease susceptibility, immunological tolerance, the magnitude of the inflammatory response, and antibody production are all modulated by anti - inflammatory cytokines (55).

LAB has been shown to control the expression of proinflammatory cytokines (such as IL-1β, IL-6, IL-10, TNF-*α*, and IFN-*γ*) and lessen inflammation in animals, including broiler chicks (56, 57). LAB generates pro-inflammatory cytokines and chemokines, release antimicrobial peptides, and activate NF-κB and MAPK signal pathways via PRRs (58). NF-κB is thought to be a significant factor in the inflammatory response and has the ability to control the expression of several cytokines, including TNF-α, IL-6, and interferon-γ (IFN-γ) (27). In our study, LAB supplementation significantly increased the levels of IL-4 and IL-2, especially in LAB-A. During the process of immune activation and regulation, IL - 2 exerts extensive up - regulative effects on not only the proliferation and differentiation of effector T cells but also on B lymphocytes. Moreover, it enables antigen dose sparing while enhancing both antigen - specific and innate immune responses (59). IL-4 has been proven to be positively correlated to IgA, which is known to control inflammation mechanisms and activate humoral immunity (60). And IgA levels have been observed to be modulated by expression of these factors (61). *Lactobacillus* strains have been reported to elicit positive immunomodulatory effects by regulating expression of key immune mediators (62). Our research suggests that dietary *L. acidophilus* treatment enhances proinflammatory and anti-inflammatory cytokine release, which in turn boosts cell-mediated immunological competence. Consequently, one of the key mechanisms underlying the immunomodulatory effects of the LAB may be the regulation of the expression of both inflammatory and anti - inflammatory cytokines, along with other immune mediators.

Lipopolysaccharide (LPS) is essential for the cell wall of gram-negative bacteria and is involved in the inflammatory reactions of hosts that *E. coli* challenges (63). Severe pathogen-induced inflammatory reactions can result in tissue damage, which is associated by elevated serum LPS levels and impaired development capacity. In this study, LAB addition reduced the level of LPS in broiler serum, which may mean the strains could alleviate the inflammation response.

*L. acidophilus* supplemented with diets decreased ADFI and F/G, reduced abdominal fat, and NH_3_ and HDL-C, which suggests that *Lactobacillus acidophilus* can improve feed conversion rate by controlling nutrient metabolism of broiler. Moreover, *L. acidophilus* increased the level of IL4 and IL-2, reduced LPS level in serum of broilers, which means that *L. acidophilus* may improve health through reducing inflammation responses of broilers.

## 5 Conclusion

In summary, this study found that the addition of *L. acidophilus* improved feed efficiency, antioxidant and immune function by regulating their nutrient metabolism and inflammatory responses. *L. acidophilus* holds significant potential as a substitute for antibiotics in broiler production, providing strong evidence for its beneficial impacts on broiler chickens.

## Data Availability

The raw data supporting the conclusions of this article will be made available by the authors, without undue reservation.
